# Occurrence of hyperoxia during iNO treatment for persistent pulmonary hypertension of the newborn: a cohort study

**DOI:** 10.1007/s00431-024-05506-6

**Published:** 2024-03-12

**Authors:** Justine de Jager, Fleur Brouwer, Jeroen Reijman, Roel L. F. van der Palen, Sylke J. Steggerda, Remco Visser, Arjan B. te Pas, Janneke Dekker

**Affiliations:** 1 Division of Neonatology, Department of Pediatrics, Leiden University Medical Center, Leiden, The Netherlands; 2 Division of Pediatric Cardiology, Department of Pediatrics, Leiden University Medical Center, Leiden, The Netherlands

**Keywords:** Hyperoxia, Persistent pulmonary hypertension, Oxygen therapy, Nitric Oxide, Newborn

## Abstract

High concentrations of oxygen are often needed to optimize oxygenation in infants with persistent pulmonary hypertension (PPHN), but this can also increase the risk of hyperoxemia. We determined the occurrence of hyperoxemia in infants treated for PPHN. Medical records of infants ≥ 34 + 0 weeks gestational age (GA) who received inhaled nitric oxide (iNO) were retrospectively reviewed for oxygenation parameters during iNO therapy. Oxygen was manually titrated to target arterial oxygen tension (PaO_2_) 10–13 kPa and peripheral oxygen saturation (SpO_2_) 92–98%. The main study outcomes were the incidence and duration of hyperoxemia and hypoxemia and the fraction of inspired oxygen (FiO_2_). A total of 181 infants were included. The median FiO_2_ was 0.43 (IQR 0.34–0.56) and the maximum FiO_2_ was 1.0 in 156/181 (86%) infants, resulting in at least one PaO_2_ > 13 kPa in 149/181 (82%) infants, of which 46/149 (31%) infants had minimal one PaO_2_ > 30 kPa. SpO_2_ was > 98% in 179/181 (99%) infants for 17.7% (8.2–35.6%) of the iNO time. PaO_2_ < 10 kPa occurred in 160/181 (88%) infants, of which 81/160 (51%) infants had minimal one PaO_2_ < 6.7 kPa. SpO_2_ was < 92% in 169/181 (93%) infants for 1.6% (0.5–4.3%) of the iNO time.

*    Conclusion*: While treatment of PPHN is focused on preventing and reversing hypoxemia, hyperoxemia occurs inadvertently in most patients.

**Table:** 

**What is Known:** *• High concentrations of oxygen are often needed to prevent hypoxemia-induced deterioration of PPHN, but this can also increase the risk of hyperoxemia.* *• Infants with persistent pulmonary hypertension may be particularly vulnerable to the toxic effects of oxygen, and hyperoxemia could further induce pulmonary vasoconstriction, potentially worsening the condition.*	
**What is New:** *• Hyperoxemia occurs in the majority of infants with PPHN during treatment with iNO.* *• Infants with PPHN spent a considerably longer period with saturations above the target range compared to saturations below the target range.*

**Supplementary Information:**

The online version contains supplementary material available at 10.1007/s00431-024-05506-6.

## Introduction

Persistent pulmonary hypertension of the newborn (PPHN) is a complication during neonatal transition characterized by a sustained elevation in pulmonary vascular resistance (PVR). The incidence of PPHN is approximately 2 per 1000 live births in late preterm and term neonates [1]. Despite appropriate therapy, PPHN is associated with a mortality rate of 10% and significant neurodevelopmental, cognitive, and hearing disabilities among survivors [1–5].

PPHN is secondary to a number of underlying causes associated with impaired relaxation of the pulmonary vasculature, such as perinatal asphyxia, infection, and meconium aspiration syndrome (MAS) [6–8]. Elevated PVR leads to decreased pulmonary blood flow (PBF), resulting in hypoxemia and acidosis. In turn, hypoxemia and acidosis cause the pulmonary vessels to constrict, thereby worsening the PPHN [8].

Postnatal increase in oxygen tension in the lungs is one of the most crucial factors to promote pulmonary vasodilatation [9]. Therefore, supplemental oxygen therapy is the mainstay in the treatment of PPHN to reduce PVR and increase oxygenation [6–8]. Inhaled nitric oxide (iNO) is a selective pulmonary vasodilator and is considered the first-line PPHN-specific therapy when oxygen therapy alone is insufficient to reduce PVR [6, 8].

High concentrations of oxygen are often needed to prevent hypoxemia-induced deterioration of PPHN, but this can also increase the risk of hyperoxemia, especially when the infant stabilizes, and PVR reduces. However, titrating oxygen therapy within the narrow therapeutic range is difficult in infants with PPHN. Infants with PPHN often experience respiratory and hemodynamic instability, which warrants caution regarding weaning off oxygen therapy.

As a result, infants with PPHN may unintentionally face an increased risk of hyperoxemia, which can cause organ injury [10]. Avoiding hyperoxemia is potentially especially relevant in the context of PPHN, as it can directly promote pulmonary vasoconstriction, further worsening the PPHN [11]. Previous translational studies have shown that there is a certain “oxygenation threshold” above which pulmonary vasodilatation is no longer enhanced and subsequent response to iNO therapy is impaired [12–15]. Moreover, reoxygenation after a period of hypoxemia may amplify or even directly cause organ injury, rendering infants with PPHN, especially those associated with perinatal asphyxia, particularly susceptible to hyperoxemia [16]. In line with this, Kilmartin et al. showed that PPHN increased the risk of death and brain injury in infants with perinatal asphyxia [17].

There is no data available regarding how often hyperoxemia occurs during PPHN treatment in infants. Therefore, we performed a retrospective cohort study to determine the occurrence of hyperoxemia in late preterm and term infants treated for PPHN.

## Methods

A retrospective study was performed including late preterm and term infants (≥ 34 + 0 weeks) admitted to the Neonatal Intensive Care Unit (NICU) at Leiden University Medical Center (LUMC) who received iNO therapy between November 2011 and June 2023. As this study aims to investigate the association between PPHN *therapy* and hyperoxemia, echocardiographic confirmation of PPHN was not considered an inclusion criterion. Exclusion criteria were as follows: iNO therapy initiated > 48 h after birth, < 2 arterial blood gasses during iNO therapy, death < 12 h after birth, and congenital malformations that affected the ability to adequately oxygenate (e.g., cyanotic congenital heart defects and lung hypoplasia).

According to the protocol of the respective NICU, iNO therapy (PrinterNO_x_, CareFusion UK 232 Ltd., Kent, UK) is indicated for infants with an arterial oxygen tension (PaO_2_) < 13.3 kPa (100 mmHg) at a fraction of inspired oxygen (FiO_2_) 1.0. iNO is started at a dose of 20 parts per million (ppm). If oxygenation improves, iNO is gradually reduced at the discretion of the respective neonatologist in decrements of 50% until 5 ppm; then, further weaning is done at a rate of 1 ppm per hour. iNO is administered through conventional or high-frequency oscillation (HFO) ventilation, with peripheral oxygen saturation (SpO_2_) limits set at 92–98% for the cardiorespiratory monitor. PaO_2_ and arterial carbon dioxide tension (PaCO_2_) targets are 10–13 kPa (75–98 mmHg) and 5–6 kPa (38–45 mmHg), respectively. Automated oxygen control is not used in PPHN. In case of inadequate improvement, sildenafil can be considered. Extracorporeal membrane oxygenation (ECMO) is used as a rescue treatment.

All study data were retrieved from two digital medical record databases (Healthcare Information X-change (HIX), Chipsoft B.V., Amsterdam, The Netherlands, and PDMS; MetaVision iMDsoft, Leiden, The Netherlands). Baseline characteristics were determined, including maternal and patient demographics, pathology underlying PPHN (categorized according to the criteria summarized in Online Resource 1), mode of ventilation, hospital course including therapeutic interventions other than oxygen and iNO, and mortality. During iNO therapy, PaO_2_ in arterial blood gasses (RapidPoint500, Siemens Healthcare, UK) were collected, as well as one-per-minute data of pre- and postductal SpO_2_ in pulse oximetry and FiO_2_. The study period varies among infants due to differences in iNO therapy duration. If an infant was transferred for ECMO, the study period was ended.

The primary study outcomes were the incidence and duration of hyperoxemia during iNO therapy, defined as a PaO_2_ > 13 kPa (98 mmHg) or SpO_2_ > 98%. Secondary outcomes included the incidence and duration of hypoxemia, defined as a PaO_2_ < 10 kPa (75 mmHg) or SpO_2_ < 92%, and the concentration and duration of oxygen therapy. The definitions for hyperoxemia and hypoxemia are based on the oxygenation target ranges for PPHN used in our NICU. Severe hyperoxemia was defined as PaO_2_ > 30 kPa (225 mmHg) as it has been associated with an increased risk of death or adverse neurodevelopmental outcome in asphyxiated infants [18]. Severe hypoxemia was defined as a PaO_2_ < 6.7 kPa (50 mmHg) or SpO_2_ < 80% as per previous studies [19, 20]. The incidences of hyperoxemia and hypoxemia were derived from collected PaO_2_ measurements, while the durations of hyperoxemia and hypoxemia were derived from one-per-minute SpO_2_ measurements.

Due to the retrospective design of the study, a convenience sample was used. Data analysis was performed using SPSS 29 (IBM SPSS Statistics). Parameters were checked for normality through visual inspection of histograms. As this observational study intends to describe oxygenation parameters during PPHN therapy, outcomes have not been tested for statistical significance. Results are presented as *n* (% of *N*) for categorical variables and mean ± SD or median (IQR) for continuous parameters.

## Results

**Fig. 1:**
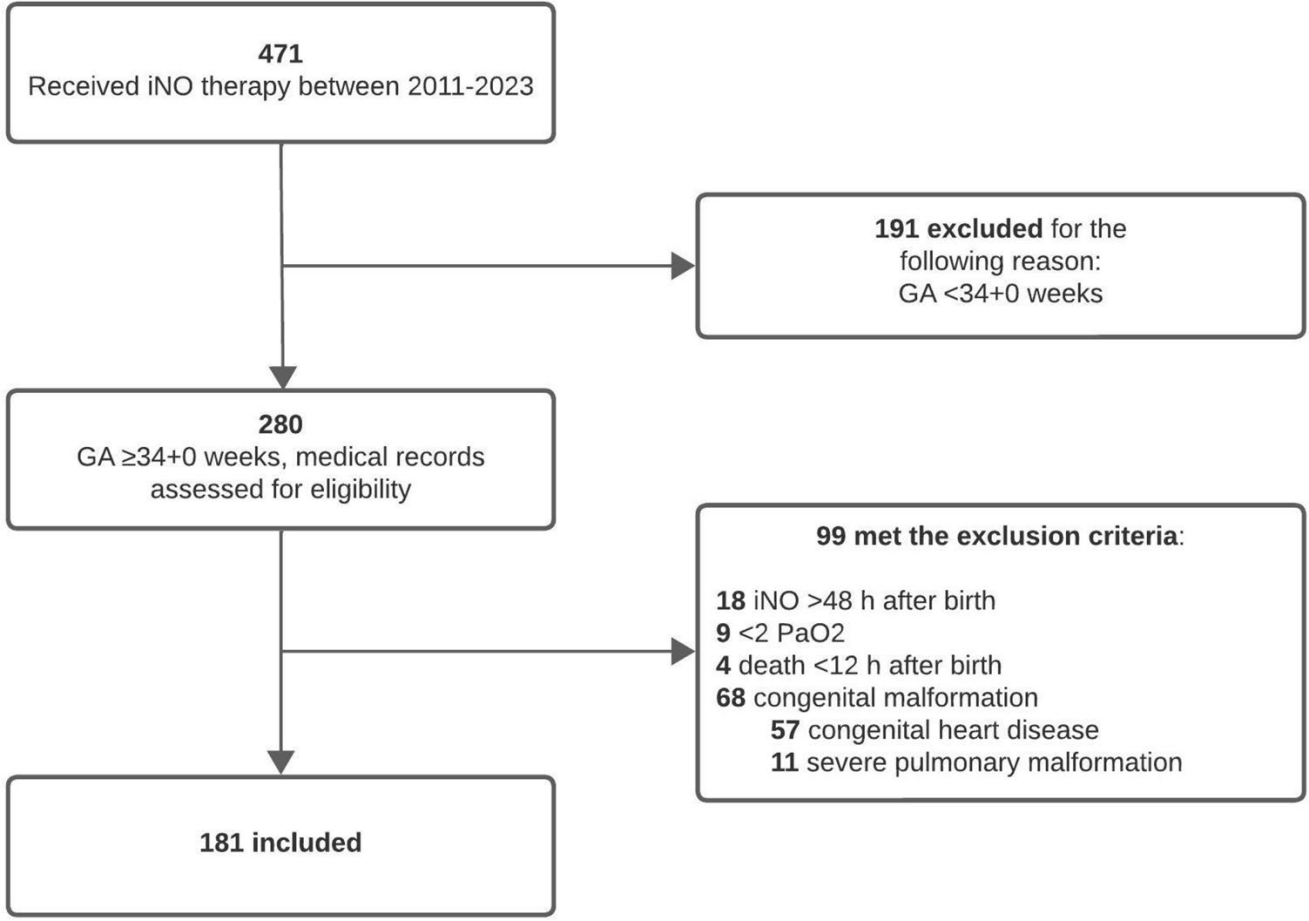
Flow chart of inclusion and exclusion criteria. The numbers in bold represent the number of infants. iNO, inhaled nitric oxide; GA, gestational age; PaO_2_, arterial oxygen tension

Between 2011 and 2023, 280 late preterm and term infants received iNO therapy in the NICU, of which 99 were excluded according to predefined criteria: 18 received iNO therapy > 48 h after birth, 9 had < 2 arterial oxygen tension measurements, 4 deceased < 12 h after birth, and 68 suffered from a congenital malformation that impaired adequate oxygenation. Thus, a total of 181 infants were included in the study (Fig. 1), with GA 40 (38–40) weeks and birth weight of 3366 ± 677 g (Table 1). The median duration of iNO therapy (study period) was 2.4 (1.5–3.6) days, with a range of 0.3 to 13.8 days. The median number of PaO_2_ samples per infant was 13 (8–19).

**Table 1:** Baseline characteristics of infants treated for PPHN

**Characteristics**	**Infants treated for PPHN (*N* = 181)**
Gestational age (complete weeks)^a^	40 (38–40)
Prematurity (GA 34 + 0–36 + 6 weeks)^b^	31 (17)
Birth weight (g)^c^	3366 (677)
Gender (male)^b^	85 (47)
Maternal parity (nulliparous)^b^	104 (58)
Type of delivery (vaginal)^b^	95 (53)
Gestation type (singleton)^b^	173 (96)
Apgar score at 5 min^a,d^	6 (4–8)
Underlying pathology^b,e^	
* Perinatal asphyxia*	29 (16)
* Infection*	28 (16)
* MAS*	4 (2)
* Combination with perinatal asphyxia, MAS and/or infection*	78 (43)
* Idiopathic*	28 (16)
* Other (pulmonary conditions, maternal disease, hydrops)*	14 (8)
Mortality during NICU admission^b^	20 (11)
Modes of invasive mechanical ventilation^b^	
* Conventional*	171 (95)
* HFO*	93 (51)
Inotropic agents^b^	163 (90)
* Dobutamine*	67 (37)
* Dopamine*	80 (44)
* Hydrocortisone*	89 (49)
* Milrinone*	94 (52)
* Noradrenaline*	109 (60)
Sedatives^b^	
* Morphine*	180 (99)
* Midazolam*	177 (98)
Atracurium^b^	21 (12)
Sildenafil^b^	5 (3)
ECMO^b^	10 (6)

*PPHN* persistent pulmonary hypertension of the newborn, *GA* gestational age, *MAS* meconium aspiration syndrome, *NICU* neonatal intensive care unit, *HFO* high-frequency oscillation, *ECMO* extracorporeal membrane oxygenation

^a^Median (IQR)

^b^*N* (%)

^c^Mean (SD)

^d^For 3/181 infants, no Apgar score was available

^e^Online Resource 1 lists the definitions used for specified pathologies

### Oxygen therapy

The median duration of oxygen therapy (FiO_2_ > 0.21) was 49 (25–83) h, constituting 97.6% (77.6–100%) of the iNO time. FiO_2_ was 1.0 in 156/181 (86%) infants for a duration of 2.2 (0.3–8.0) h per infant, representing 3.7% (0.6–13.5%) of the iNO time. The median FiO_2_ supply per infant was 0.43 (0.34–0.56).

### Hyperoxemia

**Fig. 2:**
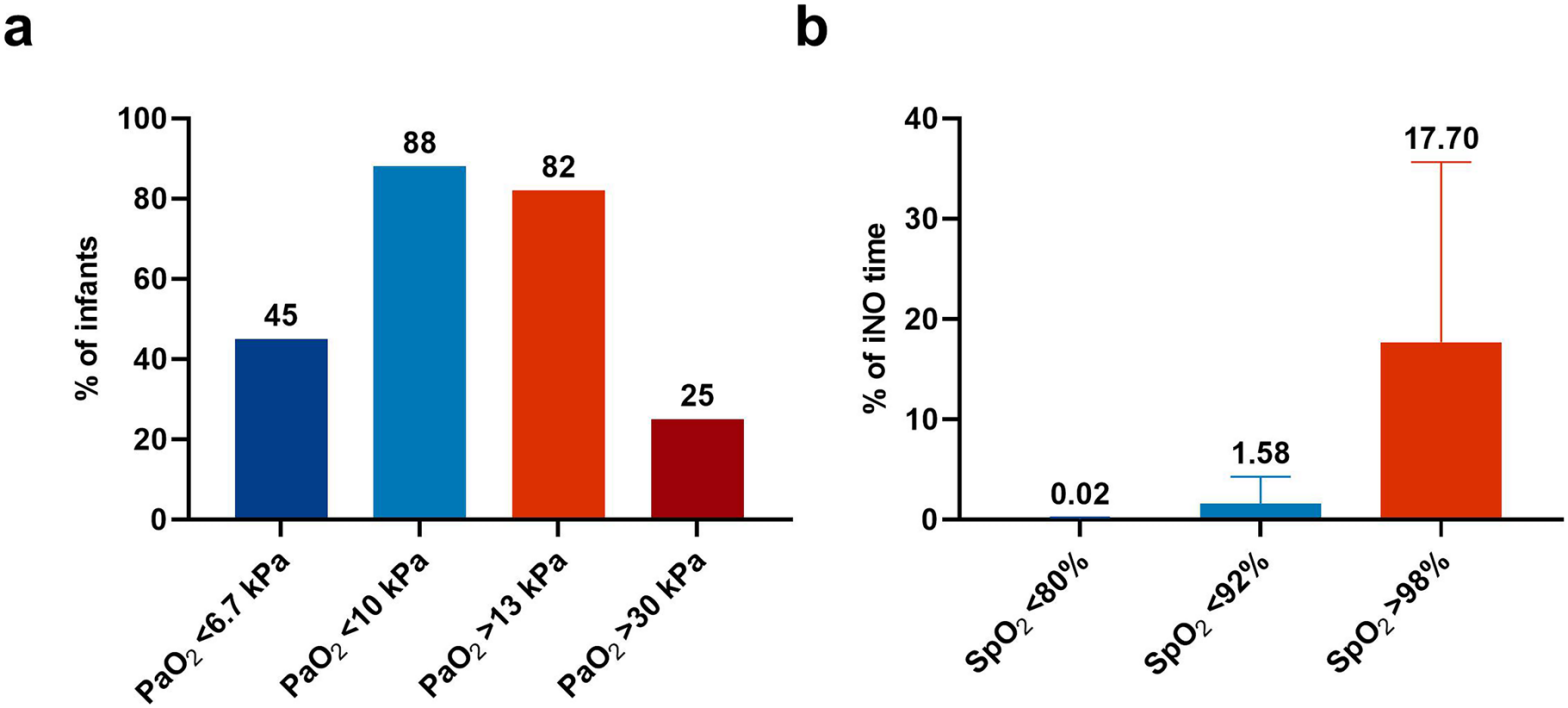
Oxygenation parameters outside the target range. **a** Proportion of infants (*N* = 181) with at least one PaO_2_ outside the target range during iNO therapy. **b** Proportion of iNO time with a SpO_2_ outside the target range. Values are presented as median (IQR). PaO_2_, arterial oxygen tension; SpO_2_, oxygen saturation (pulse oximetry); iNO, inhaled nitric oxide

In 149/181 (82%) infants, at least one PaO_2_ measurement > 13 kPa was observed and severe hyperoxemia (PaO_2_ > 30 kPa) occurred in 46/181 (25%) infants (Fig. 2). The maximum PaO_2_ during iNO therapy was 21.6 (15.4–30.3) kPa, with a range of 5.6–59.7 kPa. In 179/181 (99%) infants, SpO_2_ > 98% was observed for 17.7% (8.2–35.6%) of the iNO time (Fig. 2).

### Hypoxemia

Of the 181 infants, 160 (88%) infants had at least one PaO_2_ measurement < 10 kPa, and severe hypoxemia (PaO_2_ < 6.7 kPa) was observed in 81/181 (45%) infants (Fig. 2). The minimum PaO_2_ during iNO therapy was 6.9 (5.6–8.5) kPa, with a range of 1.4 and 22.5 kPa. In 169/181 (93%) infants, SpO_2_ < 92% was observed for 1.6% (0.5–4.3%) of the iNO time, and severe hypoxemia (SpO_2_ < 80%) occurred in 93/181 (51%) infants for 0.02% (0.00–0.22%) of the iNO time (Fig. 2).

## Discussion

In this study, we demonstrated for the first time that hyperoxemia occurs often in late preterm and term infants treated for PPHN.

A previous retrospective study by Solberg et al. in mechanically ventilated infants, irrespective of PPHN, showed that 36% of term infants with measurements of arterial blood gasses had at least one hyperoxemic PaO_2_ within 48 h, compared to 82% of the infants during treatment for PPHN in the present study [21]. Infants with PPHN constitute a subgroup of ventilated infants with an increased risk of hyperoxemia due to their high oxygen requirement, as observed in our current study in which most infants received maximum FiO_2_ for a considerable period of time. In line with this, the median FiO_2_ was higher than in the study of Solberg et al. (0.43 vs. 0.21) [21].

Remarkedly however, despite the high concentrations of oxygen therapy, the occurrence of hypoxemia was similar to that of hyperoxemia, and severe hyperoxemia (PaO_2_ < 6.7 kPa) was observed in almost half of the infants. In the acute phase, infants with PPHN typically present with severe hypoxemia, and may remain cyanotic even when exposed to a high oxygen concentration [7]. After stabilization, the infants remain labile, with even small changes causing acute pulmonary vasoconstriction and rapid clinical decompensation [22]. As a result, neonatologists are cautious in weaning FiO_2_, which increases the risk of hyperoxemia. In line with this, we found longer time periods of SpO_2_ > 98% compared to SpO_2_ < 92% within this study, indicating that the neonatologists were attentive in preventing hypoxemia but were permissive of hyperoxemia. These findings are consistent with a previous survey among 492 neonatologists in the USA that evaluated oxygen management in infants with PPHN [23]. The survey demonstrated that a significant number of neonatologists preferred to target higher SpO_2_ and/or PaO_2_ to avoid hypoxemia but did not use an upper limit of SpO_2_ and PaO_2_ to prevent hyperoxemia. Furthermore, a small but notable portion of neonatologists (6%) opted to administer 100% oxygen, irrespective of the oxygenation parameters, until they were confident that the infant had clinically stabilized [23]. The permissive attitude towards hyperoxemia is also reflected by the current significant variations in practice with respect to oxygen titration strategies and the lack of evidence-based guidelines for oxygen weaning in the management of PPHN [23, 24]. Of the 492 neonatologists from the survey, 72% did not use specific oxygen titration guidelines [23]. In line with this, in our NICU, oxygen titration is left to the discretion of the respective neonatologist. Based on the clinical perceptions of the neonatologists in this study, despite the protocol’s recommendation to avoid hyperoxemia in PPHN, it is hypothesized that titration of oxygen is initiated once PaO_2_ values are > 13 kPa to reduce the risk of rebound PPHN. However, the clinical tolerance towards hyperoxemia is challengeable in infants with PPHN as hyperoxemia can promote pulmonary vasoconstriction and add to organ injury due to reoxygenation after an hypoxemic episode. Considering the high incidence of hyperoxemia in infants with PPHN, evaluation of the risks of hyperoxemia in infants with PPHN is warranted.

In addition, re-assessment of current strategies for oxygen therapy is needed to improve the time of having oxygenation parameters within the target range in infants with PPHN. The use of standardized protocols or innovations such as automated oxygen control [25] has the potential to improve oxygen titration, but more data is required to investigate its application in infants with PPHN.

An important consideration in optimizing oxygen titration in infants with PPHN is that SpO_2_ may not accurately reflect the arterial oxygen saturation due to underlying pathologies and therapies that compromise the peripheral circulation (e.g., asphyxia, inotropic agents) and shift the oxygen-hemoglobin dissociation curve (e.g., hypothermia) [26, 27]. An alternative titration parameter could be transcutaneous oxygen tension (TcPO_2_) [28], but its accuracy in infants with PPHN requires more research. Furthermore, the optimal SpO_2_ and PaO_2_ ranges in the management of PPHN are unknown. Based on translational studies, it is recommended to maintain SpO_2_ in the low to mid-90s and PaO_2_ between 55 and 80 mmHg (7.3–10.7 kPa) [8, 11]. However, clinical studies comparing different oxygenation targets are lacking.

This study has several limitations due to its retrospective design. In infants with PPHN, preductal PaO_2_ and SpO_2_ are typically higher compared to postductal values through right-to-left shunting over the ductus arteriosus [8]. Therefore, the probability of a hyperoxic PaO_2_ and SpO_2_ is higher in preductal than postductal measurements. This study was not able to differentiate between pre- and postductal PaO_2_ and SpO_2_, which may have led to an underestimation of the occurrence of hyperoxemia. However, this would only emphasize the need to improve oxygen weaning in PPHN.

Moreover, it was not possible to compare the occurrence and duration of hyperoxemia and hypoxemia based on PaO_2_ due to sampling bias: the decision to sample an arterial blood gas depends on the clinical condition of the infant. During a period of hypoxemia or hyperoxemia, the number of PaO_2_ samples will be increased to closely monitor the infant, resulting in an increase in the occurrence of hypoxemia or hyperoxemia. Similarly, a higher number of PaO_2_ samples result in a more adequate estimation of the duration of hyperoxemia and hypoxemia. However, we were able to estimate the duration of hyperoxemia and hypoxemia in terms of SpO_2_, which is the most commonly used parameter to titrate oxygen in PPHN [23].

Furthermore, considering the wide variety in the management of PPHN [23], in particular the variation in oxygenation target ranges, the external validity of the study may be limited by its single-center design. However, the majority of neonatologists are tolerant towards hyperoxemia [23], suggesting that the trend of high occurrence of hyperoxemia demonstrated in this study may be generalizable to a broader population. Lastly, although the data collection over 11 years significantly increased the number of infants included in the study, variations in clinical practice may have occurred, resulting in a historical bias.

In conclusion, while there is an increasing awareness of oxygen toxicity, the management of PPHN remains focused on reversing hypoxemia by means of high concentrations of oxygen. As a result, hyperoxemia occurs often during manual oxygen therapy in late preterm and term infants treated for PPHN. These findings warrant clinical awareness regarding the high risk for hyperoxemia in infants with PPHN. Further research is required to improve current strategies of oxygen therapy in these infants.

### Supplementary Information

Below is the link to the electronic supplementary material.

Supplementary file1 (PDF 92 KB)

## Data Availability

The datasets generated during and/or analyzed during the current study are not publicly available due to patient privacy, but are available from the corresponding author upon reasonable request.

## Abbreviations

ECMO: Extracorporeal membrane oxygenation
FiO_2_: Fraction of inspired oxygen
GA: Gestational age
HFO: High-frequency oscillation
iNO: Inhaled nitric oxide
LUMC: Leiden University Medical Center
MAS: Meconium aspiration syndrome
NICU: Neonatal intensive care unit
PaCO_2_: Arterial carbon dioxide tension
PaO_2_: Arterial oxygen tension
PBF: Pulmonary blood flow
PPHN: Persistent pulmonary hypertension of the newborn
PPM: Parts per million
PVR: Pulmonary vascular resistance
SpO_2_: Peripheral oxygen saturation
TcPO_2_: Transcutaneous oxygen tension
