# Supplementary material for: Occurrence of hyperoxia during iNO treatment for persistent pulmonary hypertension of the newborn: a cohort study
Source: Eur J Pediatr. 2024 Mar 12;183(5):2455–61. doi: 10.1007/s00431-024-05506-6 (PMC11035448; doi:10.1007/s00431-024-05506-6)
Supplement: Supplementary file 1 — Supplementary file1 (PDF 92 KB) [file 431_2024_5506_MOESM1_ESM.pdf]

Article title: Occurrence of hyperoxia during iNO treatment for persistent pulmonary hypertension of the newborn

Journal: European Journal of Pediatrics

Author names: Justine de Jager[*1], Fleur Brouwer[1], Jeroen Reijman[1], Roel L. F. van der Palen[2], Sylke J. Steggerda[1], Remco Visser[1], Arjan B. Te Pas[1], Janneke Dekker[1]

1. Division of Neonatology, Department of Pediatrics, Leiden University Medical Center, Leiden, The Netherlands.
2. Division of Pediatric Cardiology, Department of Pediatrics, Leiden University Medical Center, Leiden, The Netherlands.

***Corresponding author:** Ms. Justine de Jager

Email: j.de_jager@lumc.nl

Pathology	Definition
Perinatal asphyxia	Oxygen deprivation during a period >10 minutes<br><br>AND <u>at least one</u> of the following criteria:<br>1. Apgar score at 5 minutes <5<br>2. Resuscitation<br>3. Ventilation (after resuscitation) >10 minutes after birth<br>4. pH <7.0*<br>5. BE <-16 mmol/L*<br>6. Lactate >10.0 mmol/L*
Meconium aspiration syndrome (MAS)	Triad of MAS:<br>1. Meconium-stained amniotic fluid (MSAF)<br>2. Respiratory distress<br>3. Confirmed by chest X-ray
Infection	Antibiotic treatment for ≥7 days
Combination	<u>At least two</u> of the following pathologies as defined above:<br>1. Perinatal asphyxia<br>2. MAS<br>3. Infection
Idiopathic	No identifiable cause
Other	All pathologies that could not be placed in the categories above

Blood from umbilical cord* *<u>or</u>*** *arterial, venous or capillary blood <1 hour after birth*
